# Supplementary material for: Effects of the COVID-19 pandemic on late postpartum women living with HIV in Kenya
Source: PLOS Glob Public Health. 2023 Mar 29;3(3):e0001513. doi: 10.1371/journal.pgph.0001513 (PMC10058168; doi:10.1371/journal.pgph.0001513)
Supplement: Attachment — Submitted filename: Response_to_reviewers_12Dec2022.docx [file pgph.0001513.s002.docx]

**Response to Reviewers**

Reviewer comments in bold.

Line numbers correspond to the word document version of the manuscript.

**Reviewer #1:**

**Overall, this manuscript was well-written and provides useful information to inform practice, policy, and future research focused on supporting pregnancy and postpartum WHIV. I would overall recommend providing a stronger argument and clarifying the reason for the focus on the late-postpartum period and to also be more specific to this population in the discussion. Specific feedback is below.**

**Introduction**

1. **This sentence is awkward: “Pregnant and breastfeeding women have also been identified as a vulnerable population during the COVID-19 pandemic given the potential risk of COVID-19 on maternal and newborn outcomes, as well as depression, anxiety and other social and psychological issues during pregnancy and postpartum.” Would recommend editing to read: “Pregnant and breastfeeding women have also been identified as a vulnerable population during the COVID-19 pandemic given the potential risk of adverse COVID-19 outcomes for this population, as well as….”**

We have edited this sentence as suggested (lines 47-48).

2. **“Yet although an estimated 1.4 million women living with HIV (WHIV) are pregnant each year globally, the unique challenges faced by this population in the late postpartum period (i.e. the period after 18 months postpartum) due to the COVID-19 pandemic have not yet been described [10].” – what is the evidence that exists re: the unique challenges of the WHIV population due to COVID-19 in pregnancy and early postpartum? You have, up to this point, only discussed general concerns of the pregnant and postpartum population due to COVID-19 – need to fill in this gap before jumping to the need to discuss late-postpartum.**

We appreciate this helpful feedback and have included the following text and additional references to enhance the focus on late postpartum WHIV (lines 48-54):

“However, to date, studies of this population have largely focused on the perinatal period or within 6-12 months postpartum, despite little evidence that pandemic-related stressors, or women’s vulnerability to them, decrease beyond these periods [10-14]. Given that an estimated 1.4 million women living with HIV (WHIV) are pregnant each year globally, the challenges faced by this population in the late postpartum period (i.e. the period after 12 months postpartum) due to the COVID-19 pandemic is important and has not yet been described [15].”

New references:

10.	Almeida M, Shrestha AD, Stojanac D, Miller LJ. The impact of the COVID-19 pandemic on women's mental health. Arch Womens Ment Health. 2020;23(6):741-8.

11.	Meaney S, Leitao S, Olander EK, Pope J, Matvienko-Sikar K. The impact of COVID-19 on pregnant womens' experiences and perceptions of antenatal maternity care, social support, and stress-reduction strategies. Women Birth. 2022;35(3):307-16.

12.	Hadjigeorgiou E, Vogazianos P, Christofi MD, et al. Experiences, concerns, and needs of pregnant and postpartum women during the Covid-19 pandemic in Cyprus: a cross-sectional study. BMC Pregnancy Childbirth. 2022;22(1):685.

13.	Chmielewska B, Barratt I, Townsend R, et al. Effects of the COVID-19 pandemic on maternal and perinatal outcomes: a systematic review and meta-analysis. Lancet Glob Health. 2021;9(6):e759-e72.

14.	Kotlar B, Gerson E, Petrillo S, Langer A, Tiemeier H. The impact of the COVID-19 pandemic on maternal and perinatal health: a scoping review. Reprod Health. 2021;18(1):10.

15.	Joint United Nations Programme on HIV/AIDS. AIDSinfo. Geneva: UNAIDS; 2020 Available from: http://aidsinfo.unaids.org/. Accessed December 21, 2022.

3. **Second paragraph – still not clear why the late postpartum period is more vulnerable to some of the listed concerns than at other times in pregnancy. Why, in particular, is this time period the focus?**

In this paragraph, we did not mean to convey that late postpartum women are *more* vulnerable than at other times during pregnancy or postpartum, but rather, that late postpartum women should not be arbitrarily considered less vulnerable or affected by the COVID-19 pandemic compared to pregnant or early postpartum women, and that research is needed to fill this gap. We have revised this paragraph as follows (lines 55-81):

“Characterizing the ways in which different sub-groups of PLHIV are affected by the COVID-19 pandemic is important, as they may have distinct vulnerabilities and needs. Such data are needed for HIV programs to effectively and efficiently implement interventions to support patients. Arbitrarily restricting interventions to women who are less than 18-24 months postpartum, for example, may exclude affected individuals beyond this period, unduly limiting the public health response. Like early postpartum women, late postpartum WHIV must also adapt to changes in autonomy and responsibilities as caregivers to young children while maintaining engagement in HIV care and adherence to antiretroviral treatment (ART). Late postpartum women may also remain vulnerable to COVID-19 control measures (e.g., ‘lockdowns’) that limit access to family, social and medical support at a time of acute need [11]. Quarantine, feelings of fatigue and isolation, fear that they or their children may become infected, and HIV- or COVID-related stigma are additional stressors that could affect late postpartum women’s mental and psychological health, making it more difficult for them to manage other parental, household and work responsibilities, or adhere to ART [12, 13].”

4. **Overall, need to strengthen argument/reasoning for targeting late-postpartum period…seem to switch between discussing affects on WHIV overall and then those who are pregnant – more consistency is needed.**

As described in the above paragraphs, we have edited the introduction in several areas to better support the rationale for focusing on last postpartum WHIV. We have also edited the manuscript text to focus on late postpartum WHIV rather more specifically than WHIV in general.

**Methods/Materials**

5. **Please define PMTCT**

We have added this description as ‘prevention of mother-to-child HIV transmission (line 138).

6. **Please more clearly define eligibility for this study – after reading the “study population” section several times, it became clear that the eligibility described in the first paragraph of that section was for a larger study and that the population for this study was recruited from there. Please define more clearly who “them” is when stating you “recruited them” and how it is separate from the parent study (also, recommended to reference the parent study if results are published).**

We have revised this section of the methods clearly define the parent study and its eligibility criteria, reference a publication describing the parent study, and define the recruitment strategy and sample size determination for the parent study (lines 135-148):

“From March 2018 to February 2019, EA-IeDEA conducted a prospective, observational study to determine the factors influencing retention in prevention of mother-to-child HIV transmission (PMTCT) care for pregnant and early postpartum WHIV who were retained in care and lost to follow-up (LTFU, defined as last clinic visit >90 days) [32, 33]. Eligibility criteria for WHIV in this parent study were: (i) age ≥18 years, (ii) pregnant or early (i.e. ≤6 months) postpartum, and (iii) enrollment at one of the following AMPATH-affiliated facilities during pregnancy: Busia District Hospital, Huruma Sub-District Hospital, Kitale District Hospital, Moi Teaching and Referral Hospital, and Uasin Gishu District Hospital. These hospitals are a mix of large and small facilities serving urban and rural populations and are representative of the various Ministry of Health facilities in the region. A convenience sample of retained WHIV were recruited by a research assistant at each facility to achieve a sample size proportional to the facility’s antenatal clinic volume. LTFU WHIV from each facility were recruited through review of the facility registers and enrolled through phone contact and community tracking.”

New reference:

33.	Humphrey J, Mwangi A, Kipchumba B, et al. A qualitative study of the barriers and enhancers to retention in care for pregnant and postpartum women living with HIV. PLOS Glob Public Health. 2021.

7. **Why were there not questions specifically related to their postpartum experience, since the target population was late-postpartum (i.e. things such as a difficult birth, morbidities postpartum, etc…could greatly impact their ability to access care, work, etc…)?**

We used a survey developed by EA-IeDEA that was designed to be implemented among different populations to facilitate future comparative analyses. We have added this sentence to the methods (lines 169-170) as well as two additional references to studies among adolescents in New York and Kenya in which this survey was implemented. We agree that further investigation of women’s postpartum experiences as they relate to the COVID-19 pandemic are needed, and we feel that this would be best accomplished through future qualitative work. We have added this point as a limitation of the study as follows (lines 396-399):

“Finally, although our study provides a rich snapshot of the pandemic’s impact on late postpartum WHIV in Kenya, programs in Kenya and other LMICs will also need to examine the pandemic’s longitudinal effects on WHIV as it evolves, as well as more deeply explore the interplay between womens’ postpartum experiences and the challenges of the COVID-19 pandemic.”

New references:

36.	Posada R, Waldman R, Chory A, et al. Longitudinal impacts of the COVID-19 pandemic on adolescents living with HIV in New York City. AIDS Care. 2022:1-5.

37.	Enane LA, Apondi E, Aluoch J, et al. Social, economic, and health effects of the COVID-19 pandemic on adolescents retained in or recently disengaged from HIV care in Kenya. PLoS One. 2021;16(9):e0257210.

**Analysis**

8. **Some more detail is needed here as based on sharing a p-value in the results, certain comparative analyses, such as chi square and or t-tests, were used.**

We have added the following text to the analysis section of the methods (lines 201-205):

“The chi-square test was used to assess associations between subgroups including those enrolled in the COVID-19 study who were retained or LTFU at enrollment in the parent study, respectively, as well as between those who had a history of public-facing work prior to the pandemic and positive mental health screening or food insecurity during the pandemic, respectively.”

**Results**

9. **Could be helpful to understand if there was any difference in concerns such as food insecurity and even positive mental health screenings between those who have work outside the home and those who didn’t prior to the pandemic, particularly knowing the association between depression and anxiety and the social distancing implemented during the pandemic.**

To address this, we have added these analyses to the methods (see above response) and results (see below, lines 311-313):

“Having public-facing work prior to the pandemic was not associated with a positive PHQ-2 or GAD-2 screen or experiencing food insecurity during the pandemic, respectively (p>0.5 for all).”

**Discussion**

10. **Would be helpful for the first paragraph to focus on providing an overall summary of the results, then move into connecting it to current literature.**

We have revised the first paragraph of the discussion to include an overall summary of the results as follows (lines 316-323):

“In this study, we describe the impact of the COVID-19 pandemic on late postpartum WHIV in western Kenya. We found that 47% of late postpartum WHIV experienced income loss and 71% experienced food insecurity during the pandemic. In addition, 96% of women reported having adequate access to antiretroviral treatment and only 3% reported difficulties refilling medications, suggesting that the HIV program’s strategies to maintain HIV service delivery during the early phase of the pandemic were effective. Still, 21% of late postpartum WHIV screened positive for depression and 8% for anxiety disorder, indicating the need for interventions to address the mental health needs of this population.”

11. **As stated in with regard to the introduction – the population of focus was WHIV in the late postpartum period, yet the discussion often focuses on WHIV overall. This type of generalization needs to be justified or more specific wording is needed to clarify you are meaning WHIV in the postpartum period.**

We have revised the text in the discussion to specifically reference ‘late postpartum WHIV’, which is the focus of the study.

12. **Per the above, consider phrasing the last sentence of the conclusion to state “spectrum of health, social and economic outcomes for late postpartum WHIV.**

We have revised the last sentence of the conclusion as suggested (lines 408-409).

**Reviewer #2**

**In this study, Humphrey et al., have attempted to understand the social, economic and health impacts of the COVID-19 pandemic on late postpartum WHIV in Kenya. This is a question of public health importance and the authors must be commended for this. I have some concerns on the validity of the estimates though.**

13. **Only 47% of all the contacted women have responded and participated in the study. This is a very sub-optimal response rate. The tendency to respond to the survey has the potential to bias the estimates in one way or the other and the most disturbing concern is that we would never know the extent and direction of such a bias.**

We agree with the reviewer that the response rate was unfortunately suboptimal. As noted in our manuscript, the low response rate is primarily attributable to difficulty recruiting WHIV who had become lost to follow-up (LTFU) (see lines 220-224). This is commonly reported among HIV studies that have sought to enroll this hard-to-reach population. We agree that the sub-optimal response rate exposes the study to potential bias. However, given the volume of literature documenting worse health outcomes among HIV clients disengaged from care compared to retained clients, it would be reasonable to infer that the estimates in our study are unlikely to be more favorable were a higher proportion of eligible LTFU WHIV enrolled in the study.

14. **Without the lack of pre-pandemic data, the burden of mental illnesses such as depression and generalized anxiety disorder attributed to the pandemic is difficult to assess. With these concerns, I’m afraid that I’m less inclined to reconcile with the estimates reported in the manuscript.**

We agree with this limitation and acknowledge it in the text by stating “the degree to which our findings reflect baseline screening rates, versus those attributable to the pandemic, is unclear” (lines 376-378). Despite this limitation, we believe that our findings are still valuable considering the limited data describing the burden of mental illness among this population in East Africa.

**Reviewer #3**

**The manuscript is technically sound and the data does support the conclusions of the study. The authors do adequately highlight the many limitations of the study and non generalizability of the results. The statistical analysis been performed appropriately and rigorously though a few areas need clarity as indicated in the additional comments below. All data underlying the findings in the manuscript is fully available. The manuscript is generally presented in an intelligible fashion and written in standard English**

15. **More detail is required by the authors on the sample size calculation for the parent study. Given the scale of the AMPATH program, how was the sample size determined? The exclusion criteria for the parent study is not indicated. These details are important as they have a direct bearing on the COVID 19 survey results.**

We have included the following text relating to the sample size determination (lines 136-150):

“From March 2018 to February 2019, EA-IeDEA conducted a prospective, observational study to determine the factors influencing retention in prevention of mother-to-child HIV transmission (PMTCT) care for pregnant and early postpartum WHIV who were retained in care and lost to follow-up (LTFU, defined as last clinic visit >90 days) [32, 33]. Eligibility criteria for WHIV in this parent study were: (i) age ≥18 years, (ii) pregnant or early (i.e. ≤6 months) postpartum, and (iii) enrollment at one of the following AMPATH-affiliated facilities during pregnancy: Busia District Hospital, Huruma Sub-District Hospital, Kitale District Hospital, Moi Teaching and Referral Hospital, and Uasin Gishu District Hospital. These hospitals are a mix of large and small facilities serving urban and rural populations and are representative of the various Ministry of Health facilities in the region. A convenience sample of retained WHIV were recruited by a research assistant at each facility to achieve a sample size proportional to the facility’s antenatal clinic volume. LTFU WHIV from each facility were recruited through review of the facility registers and enrolled through phone contact and community tracking. The study was powered to detect a 10% difference in vertical transmission between infants of retained and LTFU WHIV, assuming 40% of women were LTFU.”

16. **Under 'access and adherence to ART' section of the results, it is not clear what changes the 19% of women had to make in the way they take ART due to the pandemic. The authors need to explain what changes these were.**

We have added the following explanation to this sentence (lines 279-282):

“However, 19% of women reported having to make changes in the way they take ART because of the pandemic, such as adjusting the timing of ART dosages to accommodate work and family schedule changes, or to coincide with the availability of food.”
