# Supplementary material for: Effects of the COVID-19 pandemic on late postpartum women living with HIV in Kenya
Source: PLOS Glob Public Health. 2023 Mar 29;3(3):e0001513. doi: 10.1371/journal.pgph.0001513 (PMC10058168; doi:10.1371/journal.pgph.0001513)
Supplement: Attachment — Submitted filename: Response to Reviewers.docx [file pgph.0001513.s003.docx]

**Response to Reviewers**

(Reviewer comments in **bold** text)

**Comments to the Author**

**Reviewer 1**

**Overall, comments were addressed well. Two minor comments:**

1. **The comment regarding examining associations between having or not having a public facing job and positive PHQ-2 or GAD-2 wasn't answered quite right (comment #9), although I don't think it's essential to include this analysis. My comment stated that it would be interesting to look at associations between those who had public facing jobs versus those who did not with respect to food insecurity and/or GAD-2 and PHQ-2. i.e. was there an association between those who did NOT have a public facing job and a positive GAD-2 or PHQ-2? I would expect there might be - due to potentially more acute social isolation experienced by those who did not work. I also would think food insecurity might be more likely for that group.**

We appreciate the reviewer’s comment and agree with his/her rationale for this line of inquiry, and we regret the prior miscommunication on our part. We used the Chi-square test to assess for an association between having public-facing work and positive mental health screening or food insecurity, respectively. In the text, we wrote (lines 277-280) “Having public-facing work prior to the pandemic was not associated with a positive PHQ-2 or GAD-2 screen or experiencing food insecurity during the pandemic, respectively (p>0.05 for all).” Since these are binary variables, it is also true that NOT having a public facing job was not associated with a positive PHQ-2 or GAD-2 screen. Therefore, we have revised the text as follows:

Methods lines 169-74: “The chi-square test was used to assess associations between subgroups including those enrolled in the COVID-19 study who were retained or LTFU at enrollment in the parent study, respectively, as well as between those who those who did not have public-facing work prior to the pandemic and had positive mental health screening or food insecurity during the pandemic, respectively. SAS software was used for the analysis [40].

Results lines 277-79: “Not having public-facing work prior to the pandemic was not associated with a positive PHQ-2 or GAD-2 screen or experiencing food insecurity during the pandemic, respectively (p>0.05 for all).

2. **The first paragraph of the results could be a more general summary - i.e. instead of repeating results, what is the overall summary you want to leave the readers with? .i.e. the second sentence could say "Almost half of WHIV experienced income loss and a majority experienced food insecurity during the pandemic."**

We appreciate the reviewer’s comment and have incorporated his/her suggestions into the first paragraph of the discussion (lines 282-290):

“In this study, we describe the impact of the COVID-19 pandemic on late postpartum WHIV in western Kenya. Almost half of WHIV experienced income loss and a majority experienced food insecurity during the pandemic. In addition, nearly all women reported having adequate access to antiretroviral treatment and only 3% reported difficulties refilling medications, suggesting that the HIV program’s strategies to maintain HIV service delivery during the early phase of the pandemic were effective. Still, one in five late postpartum WHIV screened positive for depression and 8% for anxiety disorder, indicating the need for interventions to address the mental health needs of this population.”
